# Pretreatment Cancer-Related Cognitive Impairment in Hodgkin Lymphoma Patients

**DOI:** 10.3390/curroncol30100652

**Published:** 2023-10-05

**Authors:** Veronika Juríčková, Dan Fayette, Juraj Jonáš, Iveta Fajnerová, Tomáš Kozák, Jiří Horáček

**Affiliations:** 1 Center for Advanced Studies of Brain and Consciousness, National Institute of Mental Health, 25067 Klecany, Czech Republic; d.fayette@alethea.cz (D.F.); juraj.jonas@nudz.cz (J.J.); iveta.fajnerova@nudz.cz (I.F.); jiri.horacek@nudz.cz (J.H.); 2 First Faculty of Medicine, Charles University, 12108 Prague, Czech Republic; 3 Third Faculty of Medicine, Charles University, 10000 Prague, Czech Republic; tomas.kozak@fnkv.cz; 4 Faculty of Humanities, Charles University, 18200 Prague, Czech Republic; 5 Department of Clinical Hematology, University Hospital Královské Vinohrady, Šrobárova 50, 10034 Prague, Czech Republic

**Keywords:** cancer-related cognitive impairment, Hodgkin lymphoma, neuropsychology, affective distress, quality of life

## Abstract

Background: Cancer-related cognitive impairment (CRCI) is one of the most serious side effects of cancer that negatively impacts the quality of life of cancer patients and survivors. There is evidence of CRCI in Hodgkin lymphoma patients (HL); however, there is a lack of studies examining the presence of cognitive deficits before starting any treatment in HL patients. Methods: Forty adult patients (*N* = 40) newly diagnosed with HL (with no previous cancer diagnoses) and 40 healthy controls (*N* = 40) matched for age, sex, education, and premorbid intellect completed the neuropsychological battery and subjective and objective measures of affective distress and quality of life. Results: The results showed impairment in three out of six cognitive domains: verbal memory and learning, speed of processing/psychomotor speed, and abstraction/executive functions in the HL patients before the initiation of any treatment. The speed of processing/psychomotor speed domain is negatively correlated with depression. Conclusion: Cognitive deterioration in verbal memory and learning and abstraction/executive functions domains in HL patients seems to occur before the initiation of treatment independently of anxiety, depression, or physical symptoms. This suggests that HL itself may cause cognitive deficits in these cognitive domains. However, the underlying causes of CRCI still remain unclear.

## 1. Introduction

Cancer-related cognitive impairment (CRCI) is an important side effect related to decreased quality of life in oncological patients and survivors [1,2]. It is generally thought to have a typical frontal and subcortical cognitive profile and primarily involves the domains of memory, attention, executive functioning, and speed of processing [3]. These impairments are mild to moderate in severity [2], but they can have a significant impact on work capacity, ability to achieve work and educational goals, inability to drive or read, and decreased social connectedness, including occupational and social functioning, and can last for months or even years after treatment [1,4].

Persistent changes in cognitive function are frequently reported by lymphoma survivors [5,6,7], but only a few studies have focused on CRCI in Hodgkin lymphoma (HL) patients [8,9,10,11]. However, all these studies have focused on CRCI after chemotherapy or other adjuvant treatment in HL patients, reporting impairments in executive functions and memory [7], attention, working memory and planning, visual memory [8,9], verbal fluency, verbal and working memory, visual–motor coordination, and problem-solving skills [6].

Despite this, CRCI has been reported in up to 40% of patients even prior to any treatment [12,13] and demonstrated in several cancer types, including some types of hematological malignancies [2,14,15,16]. In patients with acute myeloid leukemia/myelodysplastic syndrome (AML/MDS), lower than expected scores were found in tests of memory, verbal fluency, cognitive processing speed, executive function, and fine motor dexterity [15]. Analogously, a cognitive deficit in the executive and memory domains was reported for chronic myelogenous leukemia and myelodysplastic syndrome (CML/MDS) [16]. However, there is a complete lack of studies examining the presence of cognitive deficits in HL patients before starting any treatment.

To fill this gap, we conducted a neuropsychological study on a sample of HL patients before any treatment. We aimed to map the cognitive performance of adult HL patients upon the first detection of HL diagnosis with an extensive battery of objective neuropsychological tests. The HL patients were compared with healthy controls (HC), matched for age, sex, education (in years), and premorbid intellect. Owing to the previous findings of cognitive deficits before chemotherapy in other types of cancer [15,17,18], we expected worsened cognitive functioning in the HL group compared to the HC before the initiation of any therapy. In addition, as the cognitive performance of the HL (and other oncological) patients may also be affected by psychological factors such as anxiety, depression, or physical health, we included these potential affective confounds in the assessment and analytical procedures.

## 2. Materials and Methods

### 2.1. Procedures

The study was approved by the local ethics committees of the University Hospital Kralovske Vinohrady (UHKV; EK VP 16/0/2015) in the Czech Republic. All respondents signed an informed consent to participate in the study and to process the data for research purposes. HL patients were sourced from UHKV’s Departments of Internal Medicine and Hematology. HC participants were recruited with a snowball sampling strategy through social media advertising. Participation in the research was voluntary, without compensation. All participants underwent a neuropsychological examination at the National Institute of Mental Health (NIMH) provided by a trained psychologist within a few days after establishing the diagnosis. The assessment always started at the same time in the morning (because of the potential influence of fatigue) and took about 90–120 min (including breaks) to complete. Clinically significant symptoms of distress (i.e., depressive or anxious symptoms) were operationally defined as ratings on the subjective and objective measures. 

### 2.2. Participants

The HL group consisted of 40 adult patients (55% women) with newly diagnosed Hodgkin lymphoma. Prior to surgery, adjuvant chemotherapy, and/or radiotherapy, HL patients of all histological subtypes (with the exception of lymphocyte predominance in Hodgkin’s disease) were recruited. HL patients were firstly diagnosed with Nodular sclerosis (classical) Hodgkin lymphoma (C81.1; *n* = 30; 75%), Mixed cellularity (classical) Hodgkin lymphoma (C81.2; *n* = 5; 12.5%), Lymphocyte-rich (classical) Hodgkin lymphoma (C81.4; *n* = 3; 7.5%), and Hodgkin lymphoma, unspecified (C81.9; *n* = 1; 2.5%); 1 patient’s data was insufficient (2.5%) according to ICD-10 classifications [19], and they had no previous cancer diagnoses. Exclusion criteria for HL patients included age below 18, previous history of HL or other cancer or chemotherapy, and a serious neurological or mental disorder.

The HC group consisted of 40 volunteers (55% women) meeting the exclusion criteria. Exclusion criteria for the HC included age below 18, previous history of cancer or chemotherapy, and a serious neurological or mental disorder. The HC and HL subjects were matched in sex, age, years of education, and estimated premorbid intelligence. All pairs were matched in age and education in the interval of ±3 years. For more details, see Table 1.

### 2.3. Measurements

The study subjects were administered a standardized neurocognitive battery [20] consisting of the Czech version of 13 test indices (28 subtests): the Auditory Verbal Learning Test (AVLT), Rey-Osterrieth Complex Figure Test (ROCFT), Trail Making Test (TMT), Verbal Fluency Test (VFT; phonemic and semantic), Continuous Performance Test 3 (CPT-3), four subtests of the Wechsler Adult Intelligence Scale (WAIS-III): Digits Span, Similarities, Digit Symbols–Coding, Letter-Number Sequencing, Logical Memory from Wechsler Memory (WMS-IIIa) subtest, and the Stroop Test. The Czech Reading Test [21] was used as a measure of premorbid intelligence.

All tests were standardized and validated on a healthy Czech population, designed as a Matrics-like battery, and divided into six cognitive domains: attention/vigilance, memory and learning, working memory/flexibility, verbal memory and learning, speed of processing/psychomotor speed, and abstraction/executive functions [22]. HL patients were shown to have impairments in all of these domains. [8,9,10,11]. Input variables for the Principal Component Analysis (PCA; rotation method: varimax with Kaiser normalization) utilizing a finite set of six anticipated factors were the raw scores of the various test techniques converted to z-scores [22]. The final composition of the cognitive domains was modified in light of the PCA’s findings. To decrease the dimensionality (number of subtests) and make it possible to compare the results with earlier CRCI studies, the scores from the 28 subtests were reduced by PCA to six primary cognitive domains. The composition and consistency of the cognitive domains are presented in Table 2.

To evaluate psychological distress in terms of depression and anxiety, the participants completed self-reported questionnaires: the Beck Depression Inventory (BDI-II) [23] and the Beck Anxiety Inventory (BAI-II) [24]. For complementary objective psychometrics, we applied the Hamilton Depression Rating Scale (HAM-D) [25] and the Hamilton Anxiety Rating Scale (HAM-A) [26]. Individual subjects’ quality of life was estimated using WHOQOL-BREF [27].

### 2.4. Data Processing and Statistical Analysis

Data processing and statistical analysis were processed using IBM SPSS Statistics 23 (IBM Corp., Armonk, NY, USA). The frequencies, means, and standard deviations of the study variables were examined using descriptive statistics. The main analyses focused on the differences in clinical symptoms, quality of life, and cognitive functions between the patients and the control group. The normality of the score distribution was tested by the Shapiro–Wilk Test. The data were not normally distributed among several variables, so nonparametric statistical methods were used for further analysis. The statistical significance of group differences was tested with the Mann–Whitney U Test, and the Chi-Square Test was used to test group differences in sex distribution. Based on the normative data for each of the individual test methods, the raw scores of each participant’s cognitive subtests were converted to z-scores [20]. Additionally, differences were assessed for each cognitive domain’s z-scores. To indicate statistical significance, two-sided *p*-values of 0.05 were corrected using a flexible Bonferroni–Holm procedure for multiple comparison. The relationship between performance in cognitive domains and the subjective and objective measures of affective and physical distress in the HL sample was evaluated using Spearman correlations. Linear regression models with the enter method were used to predict the HL patients’ cognitive impairments based on anxiety, depression, and physical and psychological health as predictors.

## 3. Results

The final sample included 40 HL patients and 40 HC participants. The studied groups did not differ in sex, age, years of education, or mean estimated premorbid intelligence. The demographic data for both groups and their statistical comparisons are presented in Table 1.

### 3.1. Neuropsychological Evaluations

Significant between-group differences were found in several subtests, i.e., the Auditory Verbal Learning Test (set VI and recognition); Verbal Fluency Test (semantic); Trail Making Test (parts A and B); and Similarities (Table 2). Differences in AVLT set IV and VFT semantic, on the other hand, were not considered significant because they did not pass the Bonferroni–Holm adjusted alpha threshold for multiple comparison. Other measures of the neurocognitive battery showed no significant differences between the studied groups. Analyses of the z-scores for the particular cognitive domains revealed significant differences in three out of six domains. We may consider cognitive impairment in HL patients in verbal memory and learning, speed of processing/psychomotor speed, and abstraction/executive functions (Table 3).

### 3.2. Subjective and Objective Measurement Outcomes

The HL patients scored significantly higher on both subjective and objective measures of anxiety (HAM-A, BAI-II) compared to the HC (Table 4). A significantly higher score was also identified for the objective scale of depression (HAM-D) in the HL patients, but the patients did not report increased severity of depression in the self-reported inventory (BDI-II). Significant differences between the groups were found in the quality of life of the respondents. As expected, the HL patients scored significantly lower in the domain of physical health, but they perceived their social relationships as better than the HC.

### 3.3. Correlates and Predictors of Cognitive Alterations in HL Patients

The domains of anxiety (BAI-II, HAM-A), depression (BDI-II, HAM-D), and quality of life (WHOQOL-BREF; physical and psychological health) may negatively influence cognitive performance. Spearman’s correlational analysis was used to evaluate their influence on impaired cognitive domains (Table 5). With a medium effect size, the speed of processing/psychomotor speed domain was negatively correlated with BDI-II. Therefore, we may consider the influence of depression on this domain. Positive correlations were also found between speed of processing/psychomotor speed and physical health, but the result did not hold after multiple comparison correction.

The linear regression models with the enter method were calculated to identify possible predictors of cognitive impairments in HL patients, including anxiety and depression scales and physical and psychological health scales as predictors (Table 6). The linear regression models were estimated separately for each cognitive domain (dependent variable), in which we found significant impairments (between-group differences). The regression analyses showed no significant model (Table 6).

## 4. Discussion

The main finding of this study is the confirmation of decreased cognitive functions before any treatment in patients with HL. The neuropsychological examination identified impaired cognitive performance in three out of six cognitive domains, i.e., verbal memory and learning, speed of processing/psychomotor speed, and abstraction/executive functions. In the working memory/flexibility domain, only one subtest was found to be impaired, but not the whole domain. On the contrary, our results suggest that HL patients do not have difficulty with attention/vigilance, and memory and learning (in the sense of visuospatial modality). Performance in cognitive domains was further analyzed to eliminate the influence of physical and affective symptoms related to the distress of a recent cancer diagnosis. However, only one cognitive domain, speed of processing/psychomotor speed, was found to be negatively influenced by depression. In contrast, our findings document that deterioration in the verbal memory and learning and abstraction/executive functions domains is present in HL before the initiation of treatment and occurs independently of anxiety, depression, or physical symptoms. This suggests that HL may cause cognitive deficits in these cognitive domains.

This finding is in line with memory and learning impairments identified before the initiation of therapy in breast [18,28], colorectal [29], and lung cancer patients [17,30]. Prior to treatment, patients with some hematological malignancies, such as AML/MDS and CML/MDS, have also been reported to have verbal memory and learning cognitive deficits [15,16]. The memory and learning domain deficits have also been previously identified in HL patients after treatment [8,9] but our study is the first to document them prior to treatment. Deficits in executive functions were also previously reported in HL survivors after chemotherapy [9], using the same subtest as in our study [6], so it is possible to hypothesize that some impairment was already present at the beginning of the disease and that it persists after chemotherapy. Analogously, an executive function deficit prior to treatment was also reported in AML/MDS [15] and CML/MDS [16].

Our findings of impaired speed of processing/psychomotor speed correspond with previous studies of HL patients after treatment [9,31]. We found decreased performance in both semantic verbal fluency and TMT-A subtests before treatment. Studies of HL patients have also shown impairments in TMT-A [9] and phonemic fluency [31] after treatment. Moreover, verbal fluency and cognitive processing speed in AML/MDS patients [15] and processing speed in colorectal cancer patients [29] have been shown to be affected before treatment. These findings may show evidence of a pre-treatment deficit that also persists. However, depression affected the performance in this domain in our sample. Some previous results also confirmed that fatigue, anxiety, and other emotional problems reported in HL patients negatively influence their cognitive performance [7]. Some studies, on the hand, did not associate the psychological or surgery factors with cognitive functions [1] before the initiation of therapies [4,32,33].

Not surprisingly, HL patients scored significantly higher on subjective and objective measures of anxiety and the objective scale of depression. A cancer diagnosis is associated with a high level of psychological stress in patients [34]. Anxiety and/or depression in these patients are related to uncertainty about the prognosis, planned chemotherapy or radiotherapy treatment, progressive physical deterioration, or thoughts of possible death [34]. The HL patients in our sample also reported a decrease in quality of physical health, which may be a consequence of cancer symptoms. On the other hand, the HL patients reported better social relationships compared to the HC, which may be caused by the greater need and awareness for social relations and social support in individuals facing a serious health crisis.

In conclusion, our findings of cognitive impairments in HL patients before initiation of treatment are fully consistent with the previously described pattern of cognitive impairment in CRCI (i.e., disruption of learning and memory, executive functions, and psychomotor speed). Our results suggest that cognitive impairment in HL patients could be associated with HL itself and its related symptoms. CRCI research focused on the HL population has evaluated post-chemotherapy cognitive functioning, but baseline data are completely lacking. The uniqueness of this study lies in the provision of missing pre-treatment data and in the confirmation of CRCI in HL patients. Despite that, a possible limitation of our study may be the small sample size. Our sample, on the other hand, was well-matched in terms of demographic data and corresponds to or exceeds the sample sizes of other previous studies. Moreover, we evaluated the physical health but not the fatigue itself, which was considered a possible influencing factor in CRCI in some studies [7,9,13]. Unfortunately, given the design of our study, the underlying mechanisms of CRCI in HL and other cancers remain unexplained.

Further research should elucidate whether the cognitive deficit is improved or mitigated, or, on the contrary, exacerbated by the subsequent course of HL and its treatment. In addition, longitudinal neuroimaging studies should address the structural and functional neuroanatomical changes related to cognitive deficits in HL. Clinically, further research should focus on new neuropsychological cognitive rehabilitations that may help to improve patients’ quality of life and functional and work capacities. Inspired by our results, we also suggest that cognitive functions should be monitored from the beginning of HL and complemented by cognitive training and remediation if affected. Healthcare professionals need to consider that memory impairment may interfere with a patient’s daily life functioning and may also affect their adherence to the treatment regimen. Therefore, healthcare professionals should also provide patients with remediation strategies to manage cognitive deficits if they occur at any stage of HL.

## Figures and Tables

**Table 1:** Demographic data of the study participants and between-group comparison results.

	HL Patients (*N* = 40)	Healthy Controls (*N* = 40)	Chi Square/ Mann–Whitney U Test
	Mean ± SD/Frequency	Mean ± SD/Frequency	
Sex	55% women (*n* = 22)	55% women (*n* = 22)	χ^2^ = 0.80; *p* = 0.371
Age (years)	39.14 ± 12.41 Range 19–67	38.41 ± 11.69 Range 18–65	U = 764.0; *p* = 0.729
Education (years)	14.29 ± 2.74 Range 11–21	14.74 ± 3.28 Range 11–25	U = 674.0; *p* = 0.614
Education (category)			
Elementary school	2.5% (1)	5% (2)	
Certificate of apprenticeship	20% (8)	17.5% (7)	
High school education	47.5% (19)	45% (18)	
University degree	27.5% (11)	30% (12)	
Post-doctoral education	2.5% (1)	2.5% (1)	
The Czech Reading Test (Premorbid intellect measurement)	27.77 ± 12.33	30.33 ± 11.45	U = 480.0; *p* = 0.104

**Table 2:** Raw scores of the individual test results and between-group comparison of z-scores.

Domain.	Subtests	HL Patients Mean ± SD	HC Group Mean ± SD	Mann–Whitney U Test *p*-Value
Attention/vigilance	(CPT 3-com)	51.10 ± 9.39	48.03 ± 9.02	U = 593.0; *p* = 0.094
	(CPT 3-om)	47.82 ± 6.36	47.21 ± 4.49	U = 728.5; *p* = 0.744
	(CPT 3-var)	46.42 ± 5.86	47.23 ± 9.26	U = 705.0; *p* = 0.713
	(CPT 3-det)	49.77 ± 9.16	46.67 ± 8.73	U = 639.0; *p* = 0.224
	(CPT 3-per)	48.59 ± 6.06	49.51 ± 8.62	U = 735.0; *p* = 0.792
	(CPT 3-hitsebch)	49.44 ± 8.82	48.23 ± 8.61	U = 700.5; *p* = 0.548
Visuospatial memory and learning	(ROCFT-3)	19.35 ± 6.70	20.03 ± 6.03	U = 761.0; *p* = 0.704
	(ROCFT-30)	19.56 ± 6.46	20.05 ± 6.15	U = 728.0; *p* = 0.483
Working memory/flexibility	(WAIS-III-DSp)	15.6 ± 3.47	17.55 ± 5.41	U = 655.0; *p* = 0.163
	(WAIS-III-LNS)	8.90 ± 2.69	10.33 ± 3.45	U = 610.5; *p* = 0.095
	(TMT-B)	86.30 ± 56.74	66.73 ± 28.77	U = 473.5; *p* = 0.006
	(ST-CW)	43.63 ± 10.64	43.70 ± 12.24	U = 665.0; *p* = 0.553
	(ST-IF)	5.25 ± 8.92	2.07 ± 11.93	U = 603.0; *p* = 0.159
Verbal memory and learning	(AVLT-I-V)	50.80 ± 8.88	54.10 ± 8.38	U = 596.0; *p* = 0.190
	(AVLT-B)	5.58 ± 1.78	5.85 ± 1.82	U = 759.5; *p* = 0.692
	(AVLT-VI)	10.28 ± 2.73	11.83 ± 2.77	U = 532.5; *p* = 0.010 *
	(AVLT-30)	10.50 ± 3.02	11.28 ± 2.94	U = 156.5; *p* = 0.103
	(AVLT-rec)	13.25 ± 1.72	14.23 ± 1.00	U = 526.0; *p* = 0.006
	(WMSIII-LM-imm)	42.03 ± 8.72	42.35 ± 10.60	U = 599.0; *p* = 0.478
	(WMSIII-LM-del)	26.89 ± 6.98	27.77 ± 9.20	U = 578.0; *p* = 0.343
Speed of processing/Psychomotor speed	(WAISIII-DS-C)	70.05 ± 17.81	76.63 ± 15.47	U = 610.0; *p* = 0.131
	(ST-W)	84.76 ± 12.34	88.25 ± 11.07	U = 603.5; *p* = 0.160
	(ST-C)	69.55 ± 10.24	72.40 ± 11.49	U = 638.0; *p* = 0.490
	(VFT-phonemic)	41.08 ± 10.86	44.00 ± 11.83	U = 633.0; *p* = 0.270
	(VFT-semantic)	21.77 ± 5.65	25.53 ± 6.28	U = 542.0; *p* = 0.019 *
	(TMT-A)	35.55 ± 13.82	27.15 ± 7.95	U = 421.5; *p* = 0.001
Abstraction/Executive functions	(WAIS-III-Sim)	21.08 ± 4.29	23.97 ± 4.39	U = 489.0; *p* = 0.012

Abbreviations: CPT3: continuous performance test; com: commissions; om: omissions; var: variability; det: detectability; per: perseverations; hitsebch: hit reaction time block change; ROCFT: Rey Osterrieth complex figure; 3: immediate recall after 3 min; 30: delayed recall after 30 min; WAIS-III: Wechsler adult intelligence scale; DSp: digits span; LNS: letter-number sequencing; TMT-B: Trail Making Test, part B; ST-CW: Stroop test, colors words subtest; ST-IF: Stroop test, interference score; AVLT: auditory verbal learning test; I–V: trial 1–5; VI: trial 6, 30: delayed recall after 30 min; rec: recognition; WMSIII: Wechsler memory scale; LM: logical memory; imm: immediate recall; del: delayed recall; WAIS-III: Wechsler adult intelligence scale; DS-C: digit symbols-coding; ST-W: Stroop test—words; ST-C: Stroop test—colors; VFT: Verbal Fluency Test; phonemic and semantic; TMT-A: Trail Making Test, part A; WAIS-III-Sim: Wechsler adult intelligence scale, Similarities; * results did not pass the Bonferroni–Holm adjusted alpha threshold for multiple comparison.

**Table 3:** Results of between-group differences in domains expressed in z-scores.

Cognitive Domain	HL Patients Mean ± SD	HC Group Mean ± SD	Mann–Whitney U Test *p*-Value
Attention/vigilance	0.13 ± 0.42	0.17 ± 0.46	U = 710.5; *p =* 0.617
Visuospatial memory and learning	0.23 ± 0.94	0.32 ± 0.93	U = 683.0; *p* = 0.437
Working memory/flexibility	−0.37 ± 0.61	−0.15 ± 0.88	U = 664.5; *p* = 0.337
Verbal memory and learning	−0.27 ± 0.85	0.04 ± 0.75	U = 578.5; *p* = 0.048
Speed of processing/Psychomotor speed	−0.54 ± 0.67	−0.19 ± 0.66	U = 553.0; *p* = 0.038
Abstraction/Executive functions	−0.53 ± 0.64	−0.03 ± 0.84	U = 498.0; *p* = 0.012

**Table 4:** Descriptive statistics and statistical comparisons in the subjective and objective measures of clinical symptoms.

Measures	HL Patients	HC Group	Mann–Whitney U Test
	Mean ± SD	Mean ± SD	*p*-Value
BAI-II	8.92 ± 6.97	6.51 ± 8.23	U = 513.5; *p* = 0.030
BDI-II	4.26 ± 3.73	3.31 ± 3.47	U = 625.5; *p* = 0.173
HAM-D	8.77 ± 5.86	3.74 ± 4.13	U = 367.0; *p* < 0.001
HAM-A	8.67 ± 6.23	5.28 ± 5.58	U = 488.5; *p* = 0.006
WHOQOL-BREF			
Physical health	15.2 ± 2.79	17.3 ± 1.49	U = 404.5; *p* < 0.001
Psychological health	15.9 ± 2.69	16.2 ± 1.99	U = 759.0; *p* = 0.988
Social relationships	16.8 ± 2.48	15.4 ± 2.44	U = 492.5; *p* = 0.007
Environmental Quality of Life	15.9 ± 2.08	16.5 ± 1.85	U = 650.5; *p* = 0.269

**Table 5:** Results of Spearman’s correlational analyses between subjective and objective measures of clinical symptoms and impaired cognitive domains in HL patients.

Cognitive Domain	BAI	BDI	HAM-D	HAM-A	Physical Health	Psychological Health
Verbal memory and learning	r_s_ = −0.258 *p* = 0.117	r_s_ = −0.176 *p* = 0.290	r_s_ = −0.254 *p* = 0.123	r_s_ = −0.187 *p* = 0.261	r_s_ = 0.138 *p* = 0.409	r_s_ = 0.017 *p* = 0.919
Speed of processing/Psychomotor speed	r_s_ = −0.253 *p* = 0.125	r_s_ = −0.115 *p* = 0.491	r_s_ = −0.436 ** p = 0.006	r_s_ = −0.268 *p* = 0.103	r_s_ = 0.349 *,^#^ *p* = 0.032	r_s_ = 0.240 *p* = 0.147
Abstraction/Executive functions	r_s_ = 0.045 *p* = 0.787	r_s_ = 0.134 *p* = 0.423	r_s_ = 0.038 *p* = 0.823	r_s_ = 0.093 *p* = 0.579	r_s_ = 0.084 *p* = 0.617	r_s_ = −0.155 *p* = 0.353

Note: r_s_ = result of Sperman’s correlational coefficient; * *p* < 0.05, ** *p* < 0.01. ^#^ results did not pass the Bonferroni–Holm adjusted alpha threshold for multiple comparison.

**Table 6:** Linear regression model of cognitive impairment predictors.

Cognitive Domain	*N*	R	R^2^	R^2^adj	F-Value	*p*-Value
Verbal memory and learning	38	0.415	0.172	0.012	1.076	0.398
Speed of processing/Psychomotor speed	38	0.540	0.292	0.155	2.128	0.078
Abstraction/Executive functions	38	0.391	0.153	−0.011	0.935	0.484

Note: R: simple correlation, R^2^: R-square (proportion of variance in the dependent variable, which may be predicted from the independent variables), R^2^adj: adjusted R^2^.

## Data Availability

The data presented are available on request from the corresponding author.
